# Comparative effectiveness of two first-line, ICI-based regimens for advanced HCC: a target trial emulation using an electronic medical record network

**DOI:** 10.3389/fonc.2026.1776032

**Published:** 2026-01-22

**Authors:** Chihiro Shiraishi, Miho Shigyou, Ryuichi Inoue, Toru Ogura, Susumu Kaneshige, Toshinobu Hayashi

**Affiliations:** 1 Department of Pharmaceutical Sciences for Health Crisis Management, Faculty of Pharmaceutical Sciences, Fukuoka University, Fukuoka, Japan; 2 Department of Pharmacy, Fukuoka University Hospital, Fukuoka, Japan; 3 Clinical Research Support Center, Mie University Hospital, Tsu, Mie, Japan

**Keywords:** atezolizumab, bevacizumab, durvalumab, hepatocellular carcinoma, immune-related adverse events, real-world evidence, target trial emulation, tremelimumab

## Abstract

**Introduction:**

Head-to-head comparative evidence of the relative efficacies of atezolizumab plus bevacizumab (Atezo+Bev) and tremelimumab plus durvalumab (Treme+Dur) as first-line therapies for advanced hepatocellular carcinoma (HCC) remains limited. Thus, in the present study, we compared the real-world efficacy and safety of these two modalities using a target trial emulation approach.

**Methods:**

Using the TriNetX Research Network, we identified adults (≥20 years) with HCC (ICD-10 C22.0) who initiated first-line Atezo+Bev or Treme+Dur (November 2022– November 2024). Propensity score matching (1:1) was used to balance the baseline characteristics. The primary outcome measure was overall survival (OS). Secondary outcomes included 1- or 2-year OS and organ-specific immune-related adverse events (irAEs) within 12 months, based on pre-specified ICD-10 definitions.

**Results:**

After matching, 640 patients were included in each group. Residual imbalance persisted in hepatic reserve markers (albumin, international normalized ratio, and platelet count; standardized mean differences >0.1). One-year OS was numerically higher in the Atezo+Bev group than in the Treme+Dur group (61% *vs* 55%; hazard ratio [HR], 0.806; 95% confidence interval [CI], 0.665–0.976; *p* = 0.027). Two-year OS (HR, 0.864; 95% CI, 0.723–1.031; *p* = 0.105) and overall OS showed no significant differences (median: 19.4 *vs* 19.0 months [591 *vs* 578 days]; HR, 0.886; 95% CI, 0.743–1.057; *p* = 0.179). Most irAEs were similar; however, the time-to-first hepatic irAEs favored Atezo+Bev (HR, 0.678; 95% CI, 0.487–0.943; *p* = 0.020). Notably, respiratory irAEs occurred significantly earlier in the Treme+Dur group than in the Atezo+Bev group (mean onset: 2.4 *vs* 3.2 days; *p* = 0.031).

**Conclusions:**

In this real-world target trial emulation, Atezo+Bev and Treme+Dur demonstrated broadly comparable long-term OS rates when used as first-line therapies for HCC. While baseline hepatic reserve plays an important role, the treatment regimen itself may contribute to earlier onset of hepatic and respiratory irAE. Careful monitoring for early-onset hepatic dysfunction and respiratory irAEs may be warranted in patients treated with Treme+Dur combination therapy.

## 1 Introduction

Hepatocellular carcinoma (HCC) remains a leading cause of cancer-related mortality worldwide, and a substantial proportion of patients present with or eventually develop advanced-stage disease requiring systemic therapy (1). In patients with advanced HCC, outcomes are determined not only by tumor burden but also by the severity of the underlying liver disease, which can limit treatment eligibility, affect tolerability, and strongly influence prognosis (2). Consequently, in patients with heterogeneous hepatic reserves, routine treatment selection requires balancing antitumor efficacy with safety and feasibility.

In recent years, immune checkpoint inhibitor (ICI)-based combination regimens have reshaped first-line treatment for advanced HCC. Atezolizumab plus bevacizumab (Atezo+Bev) showed improvements in overall and progression-free survival compared to sorafenib in a pivotal randomized trial and has since become a widely used first-line treatment option (3). Recently, tremelimumab plus durvalumab (Treme+Dur) demonstrated a survival benefit over sorafenib and is now incorporated into the guideline-recommended first-line therapy (4). Although both regimens have been established as standards of care, direct head-to-head comparative evidence is lacking, and cross-trial comparisons are inherently limited by differences in eligibility criteria, baseline liver function, subsequent therapies, and outcome ascertainment.

Real-world comparative studies can help address this evidence gap by evaluating the effectiveness and safety of this treatment in broader patient populations encountered in clinical practice. In practice, regimen choice is commonly shaped by hepatic reserve, endoscopic variceal ligation (EVL) history (a proxy for clinically significant portal hypertension)-related bleeding risk and anticipated immune-mediated toxicities (5). However, prior observational comparisons have often been constrained by heterogeneous comparator definitions (e.g., the inclusion of durvalumab monotherapy) and incomplete or non-organ-specific assessments of immune-related adverse events (irAEs) (6).

Target trial emulation provides a principled framework to strengthen causal interpretation using observational data by explicitly specifying the components of a hypothetical randomized trial—eligibility criteria, treatment strategies, time zero (index date), follow-up, outcomes, and estimands—and by operationalizing them in real-world datasets (7, 8). In the present study, we emulated a target trial using the TriNetX Research Network, a large multi-institutional electronic medical record (EMR) network, to compare the efficacy of Atezo+Bev with that of Treme+Dur as a first-line systemic therapy for patients with advanced HCC. The primary objective was to evaluate the overall survival (OS). The secondary objectives included assessing the 1- or 2-year OS and characterizing organ-specific irAEs, with particular attention paid to hepatic events, given the central role of baseline liver disease in this population.

## 2 Materials and methods

### 2.1 Target trial emulation framework

We conducted a targeted trial using observational data to evaluate the efficacy of Atezo+Bev *vs* Treme+Dur in patients with advanced HCC (International Classification of Diseases, Tenth Revision [ICD-10] code C22.0). This approach defines the key elements of a hypothetical RCT and replicates them in a retrospective cohort setting, with particular emphasis placed on the eligibility criteria, treatment strategies, follow-up, and analytical methods.

### 2.2 Study design and data source

We conducted a retrospective cohort study using the TriNetX Research Network, a large multi-institutional EMR network (9, 10). TriNetX provides access to de-identified EMR from approximately 250 million patients across academic medical centers, specialty practices, and community hospitals. The available data included demographics, diagnoses, procedures, medications, laboratory results, and clinical documentation. Diagnoses were identified using ICD-10-CM codes, and medications were identified using RxNorm terminology (**Supplementary Table S1**). A natural language processing network within the TriNetX Analytics Platform was also used. The database reflects racial and ethnic diversity, including White (Demographics: 2106-3), Black or African American (2054-5), Hispanic or Latino (2135-2), Asian (2028-9), and other ethnic groups.

### 2.3 Eligibility criteria

Patients aged ≥20 years with a diagnosis of HCC (ICD-10 code C22.0) were eligible. Two cohorts were defined: (1) patients who received Atezo+Bev and (2) patients who received Treme+Dur as first-line systemic treatment. All patients were treated between November 2022 and November 2024, with follow-up censored administratively on November 30, 2025. Thus, the maximum available follow-up varied according to the index date. To define first-line therapy status, we excluded patients with prior sorafenib or lenvatinib exposure before the index date. Additionally, patients in the Atezo+Bev cohort who had received prior Treme+Dur treatment and those in the Treme+Dur cohort who had received prior Atezo+Bev treatment were excluded. Patients who received subsequent systemic treatments after the index date were retained in the cohort, consistent with an observational analogue of an intention-to-treat framework. The definitions of the conditions and disease classifications are provided in **Supplementary Table S1**. The index date (day 0) was defined as the date of the initiation of Atezo+Bev or Treme+Dur administration. These criteria are summarized in **Table 1**.

**Table 1:** Target trial emulation of Atezo+Bev and Treme+Dur.

Protocol	Specification of target trials	Emulation of target trials
Eligibility criteria	- Adult patients aged ≥ 20 years - History of medical encounters with health care organizations between November 1, 2022, and November 30, 2024. - Diagnosis of HCC - Treated with either immunotherapy Atezo+Bev or Treme+Dur as the first-line treatment for HCC - No history of sorafenib or lenvatinib use before the index date - Patients in the Atezo+Bev cohort who had prior use of lenvatinib, sorafenib, or Treme+Dur were excluded, and patients in the Treme+Dur cohort who had prior use of lenvatinib, sorafenib or Atezo+Bev were excluded.	Same as for the target trials, except: - Each patient’s index date was defined as the date of the first prescription of either Atezo+Bev or Treme+Dur during the study period (November 1, 2022, through November 30, 2024)
Treatment strategies	Main analysis For the target trial comparing a Atezo+Bevinitiators and Treme+Dur initiators - First prescription of Atezo+Bev - First prescription of Treme+Dur	Same as for the target trials. Patients in each cohort were required to have at least one treatment session.
Treatment assignment	Individuals are randomly assigned to one of the comparators at baseline.	Individuals are assigned to the comparator according to the record of their medication use and assumed randomization by propensity-score matching for covariates.
Outcomes	Primary outcome - Overall survival	Same as for target trials
Follow-up	Follow-up for each individual will start at treatment assignment and end on the day of death (primary outcome), end of study, or loss to follow-up, whichever occurs first.	Same as for the target trials
Casual contrast of interest	Intention-to-treat	Observational analog to intention-to-treat
Statistical analysis	- Kaplan-Meier curve to assess the cumulative incidences of the comparator during follow-up period. - Cox proportional hazards regression to compare the risk of the preplanned outcomes daily during the follow-up period. - Subgroup analysis to assess the overall survival across prespecified patient populations stratified by demographics, disease characteristics, and treatment regimen.	Same as for the target trial, except observational analogs of intention-to-treat analyses required matching for confounding variables by propensity-score matching.

Atezo, atezolizumab; Bev, bevacizumab; Dur, durvalumab; HCC, hepatocellular carcinoma; Treme, tremelimumab.

### 2.4 Outcomes and follow-up

The primary outcome was total OS, defined as the time from the initiation of systemic therapy to death from any cause. Secondary analyses evaluated the 1- or 2-year OS and irAEs identified using ICD-10 codes categorized by organ system based on previously published literature and clinical relevance (**Supplementary Table S1**) (11). Although organ-specific irAEs were identified using ICD-10 codes, prior validation studies have demonstrated high concordance between TriNetX-coded data and clinical documentation from multi-institutional medical records (11). IrAEs were defined as newly recorded ICD-10 diagnoses after the index date that were not adjudicated for attribution or grade. The time to risk was measured from the index date until death or loss to follow-up. For irAEs, follow-up was limited to 12 months after treatment initiation to capture the early onset toxicities typically associated with ICIs. To reduce misclassification, patients with any relevant irAEs-related ICD-10 codes within 12 months before ICI initiation were excluded from the organ−specific irAE analyses. Organ system categories included cardiac, endocrine, musculoskeletal, rheumatological, hematological, hepatic, gastrointestinal, respiratory, renal, ocular, neurological, and cutaneous.

### 2.5 Covariates

Baseline covariates were grouped into the demographic, liver-related, comorbidity, metastatic, laboratory, and medication domains. Demographic variables included age, sex, body mass index (BMI, kg/m²), tobacco use, nicotine dependence, and race/ethnicity (White, Black or African American, Hispanic or Latino, and Asian). Liver-related factors included secondary esophageal varices, portal vein thrombosis, chronic viral hepatitis, alcoholic liver disease, non-alcoholic steatohepatitis (NASH), other liver diseases, other venous embolism/thrombosis, and alpha-fetoprotein (AFP) levels. Comorbidities included hypertension, dyslipidaemia, ischaemic heart disease, cerebrovascular disease, and proteinuria. The metastatic disease burden was characterized using indicators for secondary malignant neoplasms of the respiratory and digestive organs, bone and bone marrow, brain and cerebral meninges, lymph nodes, and other non-infective lymphatic disorders. Laboratory data included alanine aminotransferase (ALT, U/L), aspartate aminotransferase (AST, U/L), platelet count (×10^9^/L), total bilirubin (mg/dL), albumin (ALB, g/dL), international normalized ratio (INR), and lactate dehydrogenase (U/L). Medication-related variables included anticoagulant, platelet aggregation inhibitor, and systemic glucocorticoid use (**Supplementary Table 1**).

### 2.6 Statistical analysis

Baseline characteristics are summarized as means with standard deviations or frequencies with percentages for continuous and categorical variables, respectively. Continuous covariates (e.g. age and BMI) were entered into the models as continuous terms, whereas all other covariates were treated as categorical variables.

Propensity scores were estimated using logistic regression models that included all the baseline covariates. A 1:1 greedy nearest-neighbor matching algorithm without replacement, with a calliper width of 0.2 standard deviations of the logit of the propensity score, was applied to balance the baseline characteristics between the treatment groups. Covariate balance was assessed using absolute standardized mean differences (SMDs), with SMD < 0.1, considered indicative of acceptable balance, set as the target.

OS was analyzed in the propensity score–matched cohort using the Kaplan–Meier method. Survival probabilities at 1 or 2 years, as well as total survival, were estimated from Kaplan–Meier curves for each treatment group, while hazard ratios (HRs) with 95% confidence intervals (CIs) were obtained from Cox proportional hazards models with the treatment group (Atezo+Bev *vs* Treme+Dur) set as the main exposure. TriNetX reports Kaplan–Meier estimates among patients with follow-up information available at each horizon; denominators vary by time point. In addition, to identify independent predictors of hepatic irAEs and 1-year mortality, exploratory multivariable Cox models were fitted. Owing to platform limitations, this analysis was conducted in the overall (pre-matched) cohort, including the treatment group and other clinically relevant covariates. A two-tailed *p*-value of <0.05 was considered statistically significant. Cohort identification and statistical analyses were conducted on 4 December 2025 using the TriNetX platform query builder and analytical functions.

### 2.7 Negative control outcome

In target trial emulation, negative controls are used to detect and quantify residual bias, such as unmeasured confounding or systematic error, in observational analyses aimed at estimating causal effects. A negative control refers to an exposure, outcome, or association that is known or strongly believed not to be causally related to the exposure of interest. Therefore, any observed association indicates the presence of potential bias in the study design or analysis. Incorporating negative controls helps assess the validity of the emulation and the robustness of causal inference by determining whether the analytic methods produce spurious associations where none are expected (12). To examine residual confounding, knee osteoarthritis was selected as a negative control outcome, as it is not expected to be influenced by ICI therapy. The incidence rates and comparative risk estimates for this outcome were calculated using the same analytical methods applied to the primary outcomes (13, 14).

## 3 Results

### 3.1 Baseline characteristics

**Table 2** summarizes the baseline characteristics of the patients who received Atezo+Bev or Treme+Dur before and after propensity score matching (PSM). In the unmatched cohort, several clinically relevant imbalances were observed between the two groups. Patients receiving Treme+Dur had a higher prevalence of liver-related complications and comorbidities, including secondary esophageal varices, portal vein thrombosis, alcoholic liver disease, NASH, other liver diseases, hypertension, dyslipidaemia, ischaemic heart diseases, and proteinuria. The Treme+Dur group also had more frequent cardiovascular risk factors and showed a greater use of anticoagulant, antiplatelet agent, and glucocorticoid. In contrast, chronic viral hepatitis and Asian ethnicity were more common in the Atezo+Bev group. Laboratory data further indicated worse liver function in the Treme+Dur group, with lower ALB levels and a higher INR at baseline.

**Table 2:** Baseline characteristics.

Characteristic name	Before PSM				SMD	After PSM				SMD
	Atezo+Bev		Treme+Dur			Atezo+Bev		Treme+Dur		
	n (%)	Mean (SD)	n (%)	Mean (SD)		n (%)	Mean (SD)	n (%)	Mean (SD)	
Demographics										
Age, year	1,394 (100)	67.1 (9.9)	698 (100)	68.1 (9.8)	0.105	640 (100)	68.1 (8.9)	640 (100)	67.9 (9.9)	0.019
Male	1,083 (78)		550 (79)		0.027	507 (79)		501 (78)		0.023
BMI, kg/m^2^	934 (67)	27.4 (5.8)	544 (78)	27.8 (5.8)	0.069	509 (80)	28.1 (6.0)	492 (77)	27.6 (5.8)	0.075
Tobacco use	45 (3.2)		34 (4.9)		0.083	30 (4.7)		31 (4.8)		0.007
Nicotine dependence	207 (15)		164 (24)		0.221	140 (22)		144 (23)		0.015
Race/ethnicity										
White	654 (47)		435 (62)		0.313	416 (65)		397 (62)		0.062
Black or African American	140 (10)		104 (15)		0.147	93 (15)		87 (14)		0.027
Hispanic or Latino	120 (8.6)		83 (12)		0.108	79 (12)		78 (12)		0.005
Asian	202 (15)		65 (9.3)		0.160	57 (8.9)		62 (9.7)		0.027
Liver-related factors										
Secondary esophageal varices	181 (13)		165 (24)		0.278	135 (21)		139 (22)		0.015
Portal vein thrombosis	188 (14)		148 (21)		0.205	115 (18)		125 (20)		0.040
Chronic viral hepatitis	481 (35)		181 (26)		0.188	175 (27)		175 (27)		0.000
Alcoholic liver disease	208 (15)		152 (22)		0.178	122 (19)		133 (21)		0.043
NASH	104 (7.5)		91 (13)		0.185	74 (12)		77 (12)		0.015
Other liver diseases	668 (48)		480 (69)		0.433	431 (67)		426 (67)		0.017
Other venous embolism/thrombosis	48 (3.4)		57 (8.2)		0.203	40 (6.3)		43 (6.7)		0.019
AFP	242 (17)	25.9 (29)	204 (29)	25.6 (28)	0.013	167 (26)	25.7 (28)	185 (29)	25.4 (28)	0.010
Comorbidities										
Hypertension	766 (55)		456 (65)		0.213	414 (65)		407 (64)		0.023
Dyslipidaemia	417 (30)		276 (40)		0.203	253 (40)		242 (38)		0.035
Ischaemic heart disease	253 (18)		233 (33)		0.354	190 (30)		189 (30)		0.003
Cerebrovascular disease	93 (6.7)		61 (8.7)		0.078	47 (7.3)		50 (7.8)		0.018
Proteinuria	16 (1.1)		21 (3.0)		0.131	15 (2.3)		12 (1.9)		0.033
Metastases (Secondary Malignant Neoplasms)										
Respiratory and digestive organs	152 (11)		76 (11)		0.001	68 (11)		69 (11)		0.005
Bone and bone marrow	86 (6.2)		46 (6.6)		0.017	44 (6.9)		43 (6.7)		0.006
Brain and cerebral meninges	10 (0.7)		10 (1.4)		0.069	10 (1.6)		10 (1.6)		0.000
Lymph node	10 (0.7)		10 (1.4)		0.069	10 (1.6)		10 (1.6)		0.000
Other noninfective lymphatic disorders	18 (1.3)		10 (1.4)		0.012	10 (1.6)		10 (1.6)		0.000
Laboratory data										
ALT, U/L	1,309 (94)	53.1 (63)	683 (98)	48.9 (53)	0.072	629 (98)	54.9 (64)	625 (98)	48.9 (52)	0.103
Platelets, 10^9^/L	1,285 (92)	200.6 (114)	676 (97)	186.9 (117)	0.119	627 (98)	202.0 (117)	618 (97)	187.1 (118)	0.127
AST, U/L	1,290 (93)	78.8 (94)	682 (98)	83.2 (88)	0.049	626 (98)	83.8 (102)	624 (98)	82.5 (88)	0.014
T-Bil, mg/dL	1,251 (90)	1.6 (5.0)	648 (93)	1.6 (3.3)	0.015	603 (94)	1.4 (3.4)	600 (94)	1.6 (3.5)	0.050
ALB, g/dL	1,230 (88)	3.7 (0.6)	655 (94)	3.4 (0.6)	0.424	608 (95)	3.6 (0.6)	603 (94)	3.5 (0.6)	0.305
INR	1,202 (86)	1.1 (0.2)	614 (88)	1.2 (0.4)	0.302	569 (89)	1.2 (0.2)	565 (94)	1.2 (0.3)	0.202
LDH, U/L	318 (23)	272.2 (217)	123 (18)	256.1 (228)	0.072	118 (18)	270.0 (163)	117 (18)	257.5 (233)	0.062
Medication										
Anticoagulant	633 (45)		408 (58.5)		0.263	371 (58)		360 (56)		0.035
Platelet aggregation inhibitor	185 (13)		153 (22)		0.229	111 (17)		118 (18)		0.029
Glucocorticoid	701 (50)		463 (66)		0.330	418 (65)		415 (65)		0.010

AFP, alpha-1-fetoprotein; ALB, albumin; ALT, alanine aminotransferase; AST, aspartate aminotransferase; BMI, body mass index; INR, international normalized ratio; LDH, lactate dehydrogenase; NASH, non-alcoholic steatohepatitis; PSM, propensity score matching; SD, standard deviation; SMD, standardized mean differences; T-Bil, total bilirubin.

Before matching, 1,394 patients in the Atezo+Bev group and 698 in the Treme+Dur group met the eligibility criteria. After 1:1 propensity score matching, 640 patients were successfully matched in each treatment group. This corresponded to retention rates of 46.0% for Atezo+Bev and 91.7% for Treme+Dur, with unmatched patients excluded because suitable matches within the predefined caliper were unavailable. Most baseline characteristics were well-balanced between the groups; however, residual imbalance persisted in several laboratory surrogates of hepatic reserve, including ALB, INR, and platelet count (SMDs >0.1).

### 3.2 Overall survival in the PSM cohorts

**Figure 1:**
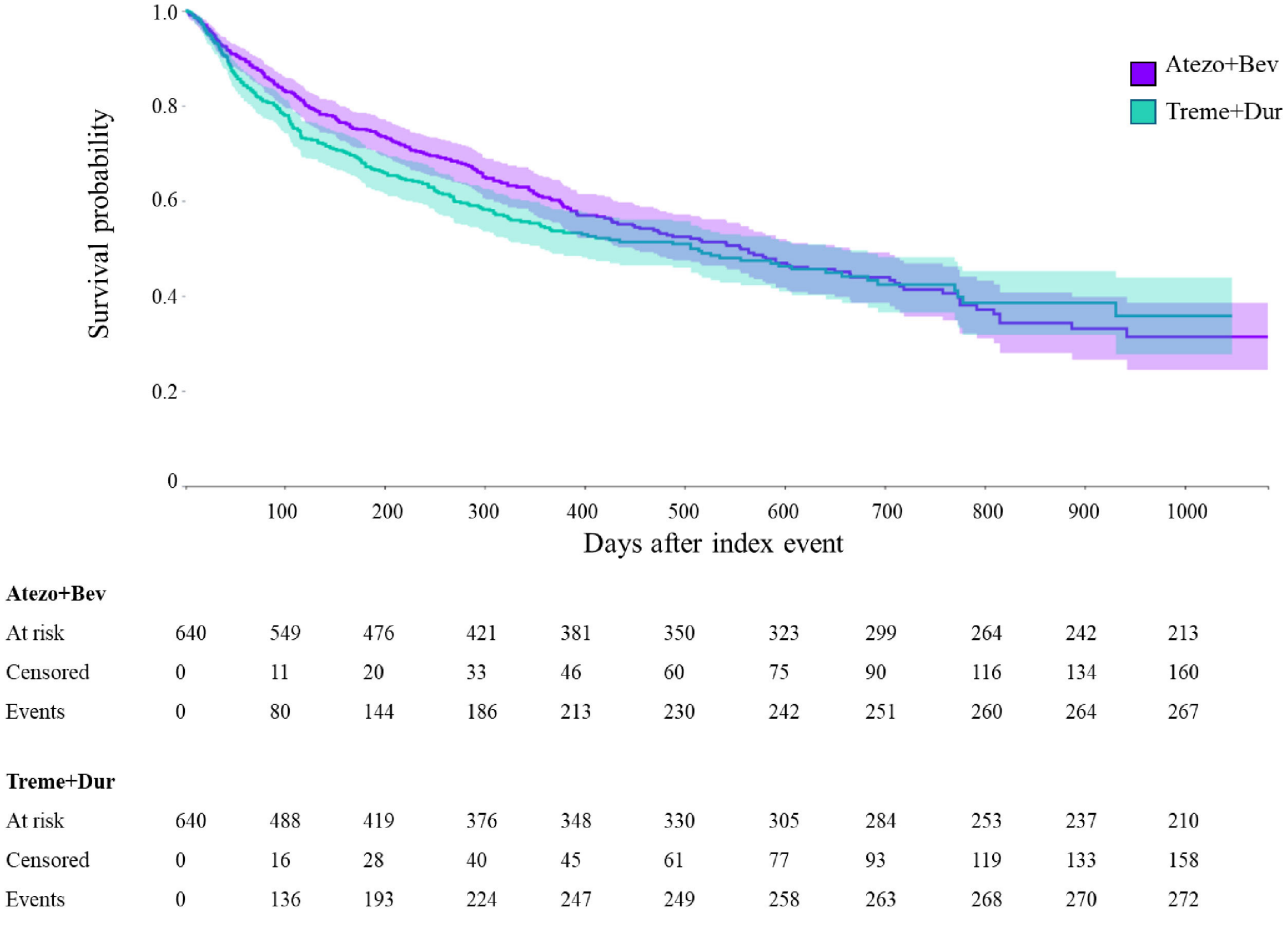
Overall survival in patients treated with Atezo+Bev or Treme+Dur. Atezo, atezolizumab; Bev, bevacizumab; Dur, durvalumab; OS, overall survival; Treme, tremelimumab. Kaplan-Meier survival curves for Atezo+Bev versus Treme+Dur in advanced HCC. Kaplan-Meier curves showing overall survival probability for patients with advanced HCC treated with Atezo+Bev (purple line) versus Treme+Dur (green line). The x-axis represents the days after treatment initiation, and the y-axis shows the survival probability. Shaded areas represent 95% CIs.

Throughout the entire follow-up period, there was no statistically significant difference in OS between the Atezo+Bev and Treme+Dur groups (median OS, 19.4 vs 19.0 months [591 *vs* 578 days]; HR, 0.886; 95% CI, 0.743–1.057; *p* = 0.179) (**Figure 1**). In the propensity score–matched cohort, the Kaplan–Meier–estimated 1-year OS was 61.1% in the Atezo+Bev group and 54.9% in the Treme+Dur group (HR, 0.806; 95% CI, 0.665–0.976; *p* = 0.027). At 2 years, the corresponding OS estimates were 43.9% and 42.9%, respectively (HR, 0.864; 95% CI, 0.723–1.031; *p* = 0.105). The survival estimates are summarized in **Table 3**.

**Table 3:** Overall survival in the PSM cohorts.

OS	Atezo+Bev	Treme+Dur	HR [95% CI]	*p*-value
Median OS, months (days)	19.4 (591)	19.0 (578)	0.886 [0.743–1.057]	0.179
OS at 1 year, %	61.1	54.9	0.806 [0.665–0.976]	0.027
OS at 2 years, %	43.9	42.9	0.864 [0.723–1.031]	0.105

Atezo, atezolizumab; Bev, bevacizumab; CI, confidence interval; Dur, durvalumab; HR, hazard ratio; OS, Overall Survival; PSM, propensity score matching; Treme, tremelimumab.

Atezo+Bev was used as the reference.

OS at 1- or 2- years are Kaplan–Meier estimates; the number at risk varies over time due to censoring.

### 3.3 Immune-related adverse events with time to first onset in the PSM cohorts

Within the first year after treatment initiation, organ-specific irAEs were recorded and categorized in the PSM cohort (**Table 4**). The incidence of most irAEs was similar between the Atezo+Bev and Treme+Dur groups. For cardiac, endocrine, musculoskeletal, hematologic, gastrointestinal, respiratory, neurological, and cutaneous events, the risk ratios were close to 1.0, and the risk differences were small, with a 95% CI crossing the null. Rheumatological, renal, and ocular irAEs were not observed in either group; therefore, effect estimates could not be calculated. Hepatic irAEs showed the largest numerical difference, occurring in 28% of patients in the Atezo+Bev group and 35% of those in the Treme+Dur group; however, this difference was not statistically significant (risk ratio, 0.798 [95% CI, 0.607–1.049]; risk difference, -7.117% [95% CI, -15.678–1.444]).

**Table:** Table 4A Immune-related adverse events in the PSM cohorts.

AE type	Atezo+Bev	Treme+Dur	RD [95% CI]	*p*-value	RR [95% CI]
Cardiac	27/611 (4.4)	29/607 (4.8)	-0.359 [-2.711–1.994]	0.765	0.925 [0.554–1.543]
Endocrine	101/444 (23)	85/443 (19)	3.560 [-1.79–8.913]	0.193	1.186 [0.917–1.533]
Musculoskeletal	61/392 (16)	49/401 (12)	3.342 [-1.470–8.154]	0.174	1.273 [0.898–1.806]
Haematological	93/462 (20)	86/398 (22)	-1.478 [-6.930–3.973]	0.595	0.932 [0.718–1.209]
Hepatic	63/224 (28)	80/227 (35)	-7.117 [-15.678–1.444]	0.104	0.798 [0.607–1.049]
Gastrointestinal	109/402 (27)	100/414 (24)	2.960 [-3.031–8.950]	0.333	1.123 [0.888–1.419]
Respiratory	135/545 (25)	130/522 (25)	-0.0134 [-5.320–5.053]	0.960	0.995 [0.807–1.226]
Neurological	75/503 (15)	59/500 (12)	3.111 [-1.095–7.316]	0.148	1.264 [0.920–1.736]
Cutaneous	111/447 (25)	117/446 (26)	-1.401 [-7.120–4.318]	0.631	0.947 [0.757–1.184]

AE, adverse event; Atezo, atezolizumab; Bev, bevacizumab; CI, confidence interval; Dur, durvalumab; PSM, propensity score matching.

RD, risk difference: RR, risk ratio; Treme, tremelimumab.

RRs are reported with 95% confidence intervals but without corresponding p-values.

Adverse events were defined as new diagnoses recorded after the index date. For each AE category, patients with a documented diagnosis of the same AE during the baseline period were excluded from the risk set; therefore, the at-risk denominator varied across AE categories.

No events were observed in either group for rheumatological, renal, or ocular AEs.

**Table:** Table 4B Time to first onset of immune-related adverse events in the PSM cohorts.

AE type	Atezo+Bev		Treme+Dur		*p*-value
	Events (n)	Days (mean [SD])	Events (n)	Days (mean [SD])	
Cardiac	27	1.5 [1.5]	29	2.0 [2.9]	0.516
Endocrine	101	4.0 [4.4]	85	3.8 [4.1]	0.765
Musculoskeletal	61	2.7 [3.4]	49	2.6 [2.1]	0.850
Haematological	93	3.0 [3.1]	86	3.1 [4.0]	0.900
Hepatic	63	2.8 [2.3]	80	2.9 [3.4]	0.847
Gastrointestinal	109	6.7 [12.8]	100	5.6 [6.0]	0.454
Respiratory	135	3.2 [3.6]	130	2.4 [2.0]	0.031
Neurological	75	2.1 [2.0]	59	2.2 [3.0]	0.755
Cutaneous	111	2.9 [3.0]	117	2.3 [2.1]	0.089

AE, adverse event; Atezo, atezolizumab; Bev, bevacizumab; CI, confidence interval; Dur, durvalumab; PSM, propensity score matching.

RD, risk difference: RR, risk ratio; SD, standard deviation; Treme, tremelimumab

Adverse events were defined as diagnoses that were newly recorded after the index date.

Patients with a documented diagnosis of the same AE during the baseline period were excluded from the risk assessment.

No events were observed in either group for rheumatological, renal, and ocular AEs.

In addition to incidence, the mean time to the first onset of irAEs was also evaluated. For most organ systems, the onset of irAEs occurred within the first few days after treatment initiation in both groups. Notably, respiratory irAEs occurred significantly earlier in the Treme+Dur group than in the Atezo+Bev group (mean onset: 2.4 *vs* 3.2 days; *p* = 0.031), whereas the timing of onset for other irAEs did not differ significantly.

**Supplementary Table 3** shows a Kaplan–Meier estimate of the time to first irAEs in the PSM cohort. In a complementary time-to-event analysis focusing on hepatic irAE, patients treated with Atezo+Bev exhibited a significantly lower hazard of hepatic irAE than those treated with Treme+Dur (HR, 0.678; 95% CI, 0.487–0.943; *p* = 0.020). The other irAEs categories did not demonstrate any significant between-group differences in the time-to-event analyses.

### 3.4 Negative control outcomes in the PSM cohort

To assess the potential unmeasured confounding factors, the incidence of knee osteoarthritis was compared between the two treatment groups. There was no significant difference in the incidence (1.8% *vs* 1.8%; HR, 0.922; 95% CI, 0.384–2.217; *p* = 0.856), providing some reassurance against large residual confounding in the primary analysis (data not shown).

### 3.5 Exploratory cox analyses in the pre-PSM cohort of hepatic events

To further explore factors associated with hepatic irAEs, we fitted a multivariate Cox proportional hazards model in the overall (pre-matched) cohort. As this analysis was conducted prior to propensity score matching, it was considered exploratory and supportive rather than a primary analysis (**Supplementary Table 4**; **Supplementary Figure 1**). In this model, treatment with Atezo+Bev was associated with a significantly lower hazard of hepatic irAEs than Treme+Dur (HR, 0.830; 95% CI, 0.715–0.963; *p* = 0.014).

Among baseline characteristics, alcoholic liver disease (HR, 1.573; 95% CI, 1.320–1.876; *p* < 0.001), fatty liver (HR, 1.373; 95% CI, 1.107–1.702; *p* = 0.004), NASH (HR, 1.887; 95% CI, 1.521–2.342; *p* < 0.001), and elevated AST levels (HR per unit increase, 1.002; 95% CI, 1.001–1.002; *p* < 0.001) were independently associated with an increased hazard of hepatic irAEs, whereas platelet count showed a modest inverse association (HR, 0.999; 95% CI, 0.998–1.000; *p* = 0.028). Other covariates, including age, sex, glucocorticoid use, chronic viral hepatitis, and ALT levels, were not significantly associated with hepatic events in this study.

### 3.6 Exploratory cox analyses in the pre-PSM cohort of 1-year mortality

In an exploratory multivariable Cox regression analysis of 1-year mortality conducted in the overall (pre-matched) cohort (**Supplementary Table 5**), treatment with Atezo+Bev was associated with a numerically lower hazard of death compared with Treme+Dur (HR, 0.865; 95% CI, 0.733–1.021; *p* = 0.087); however, this difference did not reach statistical significance. In contrast, several baseline patient characteristics were strongly associated with 1-year mortality. Markers of portal hypertension and advanced liver disease severity emerged as the most influential predictors: portal vein thrombosis (HR, 1.447; 95% CI, 1.199–1.746; *p* < 0.001) and a history of EVL (HR, 1.639; 95% CI, 1.203–2.234; *p* = 0.002) were each associated with a substantially increased risk of death.

Elevated INR was associated with more than a two-fold increase in 1-year mortality risk (HR, 2.071; 95% CI, 1.641–2.615; *p* < 0.001), whereas higher serum albumin levels were strongly protective (HR, 0.486; 95% CI, 0.424–0.556; *p* < 0.001).

Additional factors associated with increased mortality included older age (HR per year, 1.012; 95% CI, 1.004–1.021; *p* = 0.004) and the presence of secondary malignant neoplasms at other or unspecified sites (HR, 1.346; 95% CI, 1.051–1.725; *p* = 0.019). Platelet count showed a small but statistically significant positive association with mortality (HR, 1.001; 95% CI, 1.000–1.002; *p* = 0.001).

## 4 Discussion

In this large, real-world target trial using a multi-institutional EMR network, Atezo+Bev and Treme+Dur showed broadly comparable long-term OS rates when used as first-line systemic therapies for HCC. From a clinical perspective, our findings indicate that the overall incidence of irAE was broadly comparable between the two regimens, except for hepatic irAE and early-onset of respiratory irAE, which were more frequent in the Treme+Dur group. These findings suggest that treatment selection can be guided by individual patient characteristics rather than safety concerns alone.

Although direct comparisons across studies should be interpreted with caution, the survival outcomes observed in this real-world analysis appear broadly similar to those reported in the IMbrave150 and HIMALAYA randomized trials (3, 4), suggesting that comparable long-term benefits can be achieved with either regimen in clinical practice. While a numerical difference in 1-year OS was observed, this early separation likely reflects differences in baseline disease severity rather than a sustained causal effect of treatment. Although proxies of portal hypertension (prior EVL and portal vein thrombosis) were well balanced after PSM (15), modest residual imbalances persisted in markers of hepatic reserve (albumin, INR, and platelet count), which are indicative of hepatic synthetic function and associated with short-term mortality risk (**Supplementary Table 5**) (16–20), highlighting the influence of baseline clinical characteristics on early outcomes and the potential for residual confounding.

Among organ-specific irAEs, hepatic events represented the most notable safety signal in this study. Although the 12-month cumulative incidence of hepatic irAEs was numerically higher in the Treme+Dur group than in the Atezo+Bev group (35% *vs* 28%), this difference did not reach statistical significance. In contrast, the time-to-first-event analysis demonstrated a significantly lower hazard of the first hepatic irAE with Atezo+Bev, suggesting a higher cumulative incidence of early hepatic events in the Treme+Dur group rather than a difference in mean time to onset.

Immune-mediated hepatitis, which can be a fatal, manifests in several pathological patterns, including cholestatic, hepatocellular, and mixed injury (21). From a mechanistic perspective, CTLA-4 inhibitors primarily exert their immunomodulatory effects during the early phases of T-cell activation within lymphoid organs, whereas PD-1/PD-L1 inhibitors act later in peripheral tissues (22, 23). A previous study showed that combination therapies involving PD-1/PD-L1 inhibitors plus CTLA-4 inhibitors are associated with a higher risk of hepatic laboratory abnormalities, including elevation in ALT, AST, GGT, and bilirubin, compared with PD-1/PD-L1 inhibitor monotherapy, whereas no significant differences have been observed in the risk of grade 3–5 adverse events. These findings suggest that while immune-related liver injury may occur more frequently or earlier with combination therapy, severe hepatotoxicity does not necessarily increase, and treatment rechallenge may remain feasible in selected patients. Consistent with these observations, our study did not identify a higher overall burden of hepatotoxicity with Treme+Dur compared with Atezo+Bev therapy. These findings reinforce a central clinical principle in advanced HCC: patient outcomes are shaped not only by antitumor efficacy, but also—often predominantly—by the severity of the underlying liver disease and portal hypertension (24–26).

The incidence of respiratory irAEs has been reported to be <1% with Atezo+Bev and approximately 3% with Treme+Dur in patients with HCC, with most events being mild to moderate and severe cases remaining rare (27). Respiratory irAEs have been observed to occur across a broad time range, from as early as 9 days to as late as 19 months after initiation of ICI therapy. Notably, combination regimens involving CTLA-4 and PD-1/PD-L1 inhibitors are more frequently associated with earlier onset, often within the first 1–2 months, compared to monotherapy (27, 28). In our study, although the overall incidence of respiratory irAEs was comparable between the groups, patients receiving the Treme+Dur regimen experienced significantly earlier onset than those in the Atezo+Bev group (mean onset: 2.4 *vs.* 3.2 days; *p* = 0.031). Importantly, known risk factors such as smoking history and the presence of lung metastases were well balanced between the groups (29), suggesting that the difference in timing may be attributable to the treatment regimen itself. These findings have important implications for clinical practice. In particular, the earlier onset of respiratory irAEs with the STRIDE regimen underscores the need for enhanced monitoring during the first 1–3 months of therapy. Recommended strategies include baseline pulmonary assessment, frequent follow-up visits, and prompt imaging in response to new respiratory symptoms. Furthermore, previous studies have consistently shown a higher frequency of irAEs with CTLA-4–containing regimens (30), supporting the possibility that early toxicity may contribute to treatment discontinuation and potentially influence early survival differences. Prospective studies and the identification of predictive biomarkers remain critical to improving early detection and management of irAEs. Until such tools become available, clinicians should maintain a high index of suspicion and implement robust monitoring protocols during the initial phase of ICI therapy.

This study has several strengths, including an explicit target trial emulation design, a large and diverse real-world dataset encompassing both academic and community practice settings, comprehensive baseline covariate adjustment using PSM, and a prespecified organ system–based framework for irAE ascertainment. In addition, the negative control outcome analysis using knee osteoarthritis did not demonstrate any detectable between-group differences, providing reassurance against the presence of substantial residual confounding.

This study had several limitations. Firstly, residual confounding factors cannot be fully eliminated, particularly for hepatic reserve and tumor burden, due to the incomplete availability of granular staging information (e.g. BCLC), liver function scores (e.g. Child–Pugh/ALBI), radiographic response, and subsequent therapies. Second, mortality ascertainment in the EMR networks may be incomplete owing to possible out-of-network deaths or delayed reporting (31). Third, irAEs identified via ICD-10 codes may be misclassified, lack grading and attribution, and are subject to potential under-coding of mild events and documentation heterogeneity across institutions (32). Fourth, multiple outcomes were examined without any formal multiplicity adjustment, which may increase the risk of type I error, and follow-up was limited by administrative censoring, restricting evaluation of longer-term survival “tails” and late-onset toxicities. Fifth, the laboratory variables also showed substantial missingness, which may have affected matching and comparability. Sixth, owing to platform constraints, inverse probability weighting and flexible covariate adjustment were not feasible. Therefore, we employed propensity score matching, which is among the validated causal inference tools supported within the TriNetX environment. The pre-matched cohort may be subject to residual confounding and should not be interpreted as confirmatory.

## 5 Conclusion

In this real-world target trial emulation, the overall antitumor effectiveness and incidence of irAEs were broadly comparable between Atezo+Bev and Treme+Dur as first-line therapies for HCC. Although no major differences in overall toxicity burden were observed, Treme+Dur was associated with an earlier onset of hepatic adverse events, indicating that careful monitoring for early hepatic dysfunction is warranted with this regimen. Importantly, baseline liver disease severity and portal hypertension appeared to exert a greater influence on prognosis than treatment regimen itself. Future studies with longer follow-up and more granular clinical staging, liver function assessments, and treatment sequencing data are needed to clarify whether early between-regimen differences reflect causal treatment effects.

## Data Availability

The datasets presented in this article are not readily available because the dataset used in this study was obtained from the TriNetX research network and is subject to data use agreements and privacy regulations. The data are de-identified and cannot be shared publicly. Access to the dataset is restricted to licensed users of the TriNetX platform and cannot be made available through a public repository or upon request. Requests to access the datasets should be directed to https://trinetx.com/products/real-world-datasets/.
