# Supplementary material for: Comparative effectiveness of two first-line, ICI-based regimens for advanced HCC: a target trial emulation using an electronic medical record network
Source: Front Oncol. 2026 Jan 22;16:1776032. doi: 10.3389/fonc.2026.1776032 (PMC12872483; doi:10.3389/fonc.2026.1776032)
Supplement: Supplementary file 1 [file DataSheet1.docx]

**Supplemental Table 1**. Definitions of conditions and diseases

| BMI | TNX Curated 9083 |
| --- | --- |
| Tobacco use | Z72.0 Tobacco use |
| Nicotine dependence | F17 Nicotine dependence |
| White | White (Demographics: 2106-3) |
| Black or African American | Black or African American (Demographics: 2054-5) |
| Asian | Asian (Demographics: 2028-9) |
| Hispanic or Latino | Hispanic or Latino (Demographics: 2135-2) |
| Secondary oesophageal varices | I85.1 Secondary esophageal varices |
| Portal vein thrombosis | I81 Portal vein thrombosis |
| Chronic viral hepatitis | B18 Chronic viral hepatitis |
| Alcoholic liver disease | K70 Alcoholic liver disease |
| Nonalcoholic steatohepatitis | K75.81 Nonalcoholic steatohepatitis (NASH) |
| Other liver diseases | K76 Other diseases of liver |
| Other venous embolism/thrombosis | I82 Other venous embolism and thrombosis |
| Liver cancer | C22 Liver cell carcinoma  ICD-O Liver and intrahepatic bile ducts |
| Autoimmune hepatitis | K75.4 Autoimmune hepatitis |
| Hypertension | I10 Essential (primary) hypertension  I11 Hypertensive heart disease  I12 Hypertensive chronic kidney disease  I13 Hypertensive heart and chronic kidney disease  I15 Secondary hypertension |
| Dyslipidaemia | E78 Disorders of lipoprotein metabolism and other lipidemias |
| Cerebrovascular disease | I60 Nontraumatic subarachnoid hemorrhage  I61 Nontraumatic intracerebral hemorrhage  I62 Other and unspecified nontraumatic intracranial hemorrhage  I63 Cerebral infarction  I65 Occlusion and stenosis of precerebral arteries, not resulting in cerebral infarction  I66 Occlusion and stenosis of cerebral arteries, not resulting in cerebral infarction  I67 Other cerebrovascular diseases  I68 Cerebrovascular disorders in diseases classified elsewhere  I69 Sequelae of cerebrovascular disease |
| Proteinuria | R80 Proteinuria |
| Ischaemic heart disease | I20 Angina pectoris  I21 Acute myocardial infarction  I22 Subsequent ST elevation (STEMI) and non-ST elevation (NSTEMI) myocardial infarction  I23 Certain current complications following ST elevation (STEMI) and non-ST elevation (NSTEMI) myocardial infarction (within the 28-day period)  I24 Other acute ischemic heart diseases  I25 Chronic ischemic heart disease |
| Cardiomyopathy | I42 Cardiomyopathy |
| Heart failure | I50 Heart failure |
| Secondary malignant neoplasm (Respiratory and digestive organs) | C78.0 Secondary malignant neoplasm of respiratory and digestive organs |
| Secondary malignant neoplasm of other and unspecified sites | C79.0 Secondary malignant neoplasm of other and unspecified sites |
| Secondary malignant neoplasm (Bone and bone marrow) | C79.5 Secondary malignant neoplasm of bone and bone marrow |
| Secondary malignant neoplasm (Brain and cerebral meninges) | C79.3 Secondary malignant neoplasm of brain and cerebral meninges |
| Secondary lymph node neoplasm | C77.9 Secondary and unspecified malignant neoplasm of lymph node, unspecified |
| Endoscopic variceal ligation (EVL) | 43244 Esophagogastroduodenoscopy, flexible, transoral; with band ligation of esophageal/gastric varices |
| Other noninfective lymphatic disorders | I89 Other noninfective disorders of lymphatic vessels and lymph nodes |
| Alanine aminotransferase | TNX Curated 9044 |
| Platelets | TNX Curated 9020 |
| Aspartate aminotransferase | TNX Curated 9047 |
| Total bilirubin | TNX Curated 9050 |
| Albumin | TNX Curated 9045 |
| INR | TNX Curated 9032 |
| Lactate dehydrogenase | TNX Curated 9052 |
| Alpha-1-Fetoprotein | LG5597-2 |
| Anticoagulant | BL110 Anticoagulants |
| Platelet aggregation inhibitor | BL117 Platelet aggregation inhibitors |
| Glucocorticoid | VA H02AB Glucocorticoids |
| Atezolizumab | RxNorm 1792776  HCPCS J9022, C9483  ICD-10-PCS XW033D6 |
| Bevacizumab | RxNorm 253337  HCPCS Q5129, J9035, C9257, Q5107, Q5118, Q5126 |
| Tremelimumab | RxNorm 2619313, OMOP5178384  HCPCS C9492, J9347 |
| Durvalmab | RxNorm 1919503  HCPCS J9173 |
| Regorafenib | RxNorm 1312397 |
| Ramucirumab | RxNorm 1535922  HCPCS J9308, C9025 |
| Nivormab | RxNorm 1597876  HCPCS J9299, J9298, C9453 |
| Ipilimumab | RxNorm 1094833  HCPCS J9228 |
| Sorafenib | RxNorm 495881 |
| Cabozantinib | RxNorm 1363268 |
| Lenvatinib | RxNorm 1603296 |

**Supplemental Table 2**. ICD-10 codes used to identify immune-related adverse events

| Organ System Event Category | Organ System Event Category | ICD-10 |
| --- | --- | --- |
| 1. Cardiac | Myocarditis | I40, I41, I51.4, I51.8, I51.9 |
|  | Pericarditis | I30, I31 |
| 2. Endocrine | Adrenal insufficiency | E27.1, E27.2, E27.3, E27.4 |
|  | Hyper or Hypo-parathyroidism | E20, E21 |
|  | Hyperthyroidism | E05 |
|  | Hypophysitis or Polyglandular autoimmune (PGA) syndrome (Hypophysitis or PGA) | E23.3, E23.6, E23.7, E31.0 |
|  | Other adrenal gland disorders (Other AGDs) | E26, E25.8, E25.9, E27.0, E27.5, E27.8, E27.9 |
|  | Hypothyroidism | E03.2, E03.8, E03.9 |
|  | Thyroiditis | E06, E05.8, E05.9, E07.8, E07.9 |
|  | Type I Diabetes | E09, E10, E13 |
|  | Hyperglycemia | R73. 9 |
| 3. Musculoskeletal | Arthralgias or Myalgias | M79.1 |
|  | Arthritis | M13, M17.0, M17.1, M17.4, M17.5, M17.9, M19, M25 |
|  | Arteritis | I77.6 |
| 4. Rheumatological | Psoriatic Arthritis | L40.5 |
|  | Vasculitis | M30.0, M30.1, M31.0, M31.2, M31.3, M31.4, M31.5, M31.6 |
|  | Lupus | M32 |
|  | Sclerosis | M34 |
|  | Sicca syndrome | M35.0 |
|  | Connective tissue disease | M35.5, M35.8, M35.9 |
|  | Inflammatory myositis | G72.49 |
|  | Rheumatoid arthritis | M05, M06 |
|  | Polymyalgia rheumatica | M35.3 |
|  | Reiter's disease | M02.3 |
|  | Paget's disease of bone | M88 |
|  | Sarcoidosis | D86 |
| 5.Haematological | Anemia | D59, D60, D61, D64.2, D64.3, D64.8 |
|  | Leukopenia | D76.1, D76.2, D76.3, D72.81, D72.89, D72.9 |
|  | Eosinophilia | D72.1 |
|  | Neutropenia | D70.2, D70.4, D70.8, D70.9 |
|  | Thrombocytopenia | D69.3, D69.41, D69.42, D69.49, D69.5, D69.6, D69.8, D69.9 |
| 6. Hepatic | Other chronic hepatitis, unspecified | K73.8, K73.9 |
|  | Nonspecific reactive hepatitis | K75.2 |
|  | Autoimmune hepatitis | K75.4 |
|  | Inflammatory liver disease, unspecified | K75.89, K75.9 |
|  | Hepatitis | K71, R74.8, R94.5, K76.8, K76.9 |
| 7. Gastrointestinal | Inflammatory bowel disease (IBD) | K50, K51, K52.1, K52.3, K52.89, K52.9 |
|  | Eosinophilic gastritis and colitis | K52.81, K52.82 |
|  | Microscopic colitis | K52.83 |
|  | Esophagitis | K20.9 |
|  | Gastroduodenitis | K29.0, K29.1, K29.4, K29.5, K29.6, K29.7, K29.9 |
|  | Duodenitis | K29.8 |
|  | Celiac disease | K90.0 |
|  | Cholecystitis | K81 |
|  | Diarrhea | R19.7 |
|  | Pancreatitis | K85 |
|  | Xerostomia | R68.2 |
| 8. Respiratory | Pneumonitis | J18.8, J18.9, J70.2, J70.3, J70.4, J70.8, J70.9, J84.11, J84.89, J84.9 |
|  | Bronchiolitis | J21.9 |
| 9. Renal | Acute kidney injury (AKI) | N00, N01, N04, N05, N06, N08, N14.1, N14.2, N14.4, N17 |
|  | Kidney failure, unspecified | N19 |
| 10. Ocular | Uveitis | H20 |
|  | Ophthalmoplegia | H49.3, H49.4 |
|  | Other eye movement disorders (Other EMDs) | H51 |
|  | Keratitis | H16 |
|  | Conjunctivitis | H10, H11 |
|  | Inflammation of eyelid | H01 |
|  | Optic neuritis | H46 |
|  | Vitritis | H43 |
|  | Diplopia | H53.2 |
|  | Chorioretinal inflammation | H30 |
| 11. Neurological | Meningitis | A87, G03 |
|  | Neuritis | G60.3, G60.8, G60.9, G61.1, G62.9, G90, M79.2 |
|  | Myasthenia gravis | G70.0 |
|  | Myasthenic syndrome | G73.3 |
|  | Lambert-Eaton syndrome | G73.1, G70.80, G70.81 |
|  | Toxic myoneural disorders | G70.1 |
|  | Encephalomyelitis | G04.0, G04.2, G04.8, G04.90, G04.91, G92 |
|  | Bell’s palsy | G51.0 |
|  | Guillain-Barré syndrome | G61.0 |
|  | Drug-induced polyneuropathy | G62.0 |
|  | Polyneuropathy due to other toxic agents | G62.2 |
|  | Inflammatory neuropathy, unspecified | G61.8, G61.9 |
|  | Multiple sclerosis | G35 |
|  | Other demyelinating diseases | G36, G37 |
|  | Other disorders of nervous system | G96.8, G96.9, G98, G99 |
|  | Dysphagia | R13.1 |
|  | Disturbance of skin sensation | R20 |
| 12. Cutaneous | Acne | L70 |
|  | Psoriasis | L40.0, L40.1, L40.4, L40.8, L40.9 |
|  | Parapsoriasis | L41.0, L41.1, L41.3, L41.4, L41.5, L41.8, L41.9 |
|  | Pityriasis rosea | L42 |
|  | Pityriasis rubra pilaris | L42 |
|  | Rash or Pruritus | R21, L29 |
|  | Urticaria | L50.0, L50.1, L50.3, L50.8, L50.9 |
|  | Drug hypersensitivity of skin | L27.0, L27.1, L27.8, L27.9 |
|  | Drug Rash with Eosinophilia and Systemic Symptoms (DRESS) | D72.12 |
|  | Exfoliative dermatitis | L26 |
|  | Alopecia | L63, L64, L65, L66 |
|  | Dermatomyositis | M33, D49.9 |
|  | Vitiligo | L80 |
|  | Hyperpigmentation | L81.4, L81.8 |
|  | Dyspigmentation | L81.9 |
|  | Vasculitis skin | L95, D69.0 |
|  | Grover's disease | L11.1 |
|  | Scleroderma | L94.0, L94.1, L94.2, L94.3 |
|  | Follicular disorders | L11.0, L72, L73, L87.0 |
|  | Sarcoidosis skin | L92.9 |
|  | Hyperkeratosis | L85.9 |
|  | Keratoacanthoma | L85.8 |
|  | Seborrheic dermatitis | L21 |
|  | Actinic keratosis | L57.0 |
|  | Other specified dermatoses | L98.8 |
|  | Eczema | L20, L23, L24, L25, L30.8, L30.9 |
|  | Photosensitivity | L56.0, L56.1, L56.8, L56.9 |
|  | Granuloma annulare | L92.0 |
|  | Hyperhidrosis | L74.519, L74.52, R61 |
|  | Onycholysis | L60.1 |
|  | Xerosis | L85.3 |
|  | Anhidrosis | L74.4 |
|  | Erythematous conditions | L53 |
|  | EM/SJS/TEN | L51 |
|  | Rosacea | L71 |
|  | Lupus erythematosus | L93 |
|  | Panniculitis | M54.0, M79.3 |
|  | Erythema nodosum | L52 |
|  | Lichen planus | L43, L44.3, L66.1 |
|  | Mucositis | K12, K13.7 |
|  | Neutrophilic dermatitis | L88, L98.2 |
|  | Bullous dermatitis | L01.03, L10, L12, L13, L14 |

ICD-10: The International Classification of Diseases, Tenth Revision; NOS: not otherwise specified; SJS: Stevens-Johnson syndrome; TEN: toxic epidermal necrolysis

**Supplemental Table 3**. Time to First Occurrence of Immune-Related Adverse Events: Hazard Ratios from Cox Proportional Hazards Model

|  | HR [95% CI] | *p*-value |
| --- | --- | --- |
| Cardiac | 0.809 [0.479–1.367] | 0.428 |
| Endocrine | 1.032 [0.773–1.377] | 0.834 |
| Musculoskeletal | 1.106 [0.759–1.611] | 0.600 |
| Haematological | 0.799 [0.596–1.072] | 0.134 |
| Hepatic | 0.678 [0.487–0.943] | 0.020 |
| Gastrointestinal | 1.000 [0.762–1.312] | 1.000 |
| Respiratory | 0.861 [0.676–1.095] | 0.221 |
| Neurological | 1.125 [0.800–1.583] | 0.499 |
| Cutaneous | 0.823 [0.634–1.067] | 0.140 |

CI, confidence interval; HR, hazard ratio

Adverse events were defined as diagnoses that were newly recorded after the index date.

No events were observed in either group for rheumatological, renal, and ocular AEs.


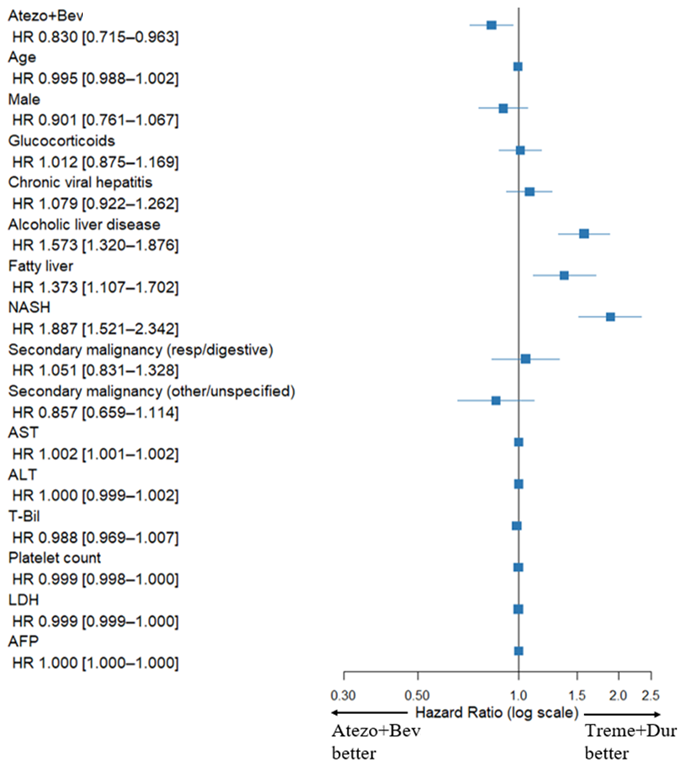


**Supplemental Figure 1**. Forest Plot from Cox Regression Analysis of Hepatic Events

Forest plot of hepatic events comparing Atezo+Bev versus Treme+Dur across patient subgroups. The x-axis shows the HR scale. The diamonds represent the HRs, and the horizontal lines represent the 95% CIs. HR = 1 represents the line of no effect.

HR < 1 favours Atezo+Bev.

AFP, alpha-1-fetoprotein; ALT, alanine aminotransferase; Atezo, atezolizumab; AST, aspartate aminotransferase; Bev, bevacizumab; Dur, durvalumab; LDH, lactate dehydrogenase, NASH, non-alcoholic steatohepatitis; T-Bil, total bilirubin; Treme, tremelimumab

**Supplemental Table 4**. Cox Regression Analysis of Hepatic Events

| Covariate | HR (95% CI) | *p*-value |
| --- | --- | --- |
| Atezo+Bev (*vs.* Treme+Dur) | 0.830 [0.715–0.963] | 0.014 |
| Age | 0.995 [0.988–1.002] | 0.161 |
| Male | 0.901 [0.761–1.067] | 0.227 |
| Glucocorticoids | 1.012 [0.875–1.169] | 0.877 |
| Chronic viral hepatitis | 1.079 [0.922–1.262] | 0.345 |
| Alcoholic liver disease | 1.573 [1.320–1.876] | < 0.001 |
| Fatty liver | 1.373 [1.107–1.702] | 0.004 |
| NASH | 1.887 [1.521–2.342] | < 0.001 |
| Secondary malignant neoplasm (Respiratory and digestive organs) | 1.051 [0.831–1.328] | 0.680 |
| Secondary malignant neoplasm (other/unspecified) | 0.857 [0.659–1.114] | 0.248 |
| AST | 1.002 [1.001–1.002] | < 0.001 |
| ALT | 1.000 [0.999–1.002] | 0.705 |
| T-Bil | 0.988 [0.969–1.007] | 0.227 |
| Platelet count | 0.999 [0.998–1.000] | 0.028 |
| LDH | 0.999 [0.999–1.000] | 0.220 |
| AFP | 1.000 [1.000–1.000] | 0.596 |

Atezo, atezolizumab; AFP, alpha-1-fetoprotein; ALT, alanine aminotransferase; AST, aspartate aminotransferase; Bev, bevacizumab; CI, confidence interval; Dur, durvalumab; HR, hazard ratio; LDH, lactate dehydrogenase, NASH, non-alcoholic steatohepatitis; T-Bil, total bilirubin; Treme, tremelimumab

Atezo+Bev was used as the reference.

**Supplemental Table 5**. Cox regression analysis of 1-year mortality

| Covariate | HR (95% CI) | *p*-value |
| --- | --- | --- |
| Atezo+Bev (*vs*. Treme+Dur) | 0.865 [0.733–1.021] | 0.087 |
| Age | 1.012 [1.004–1.021] | 0.004 |
| Male | 0.979 [0.813–1.179] | 0.823 |
| Glucocorticoids | 0.973 [0.832–1.139] | 0.733 |
| AFP | 1.000 [1.000–1.000] | 0.288 |
| Ischaemic heart disease | 1.067 [0.885–1.285] | 0.498 |
| Cardiomyopathy | 0.925 [0.528–1.621] | 0.786 |
| Heart failure | 1.105 [0.842–1.451] | 0.472 |
| Portal vein thrombosis | 1.447 [1.199–1.746] | <0.001 |
| Proteinuria | 1.028 [0.597–1.770] | 0.920 |
| Endoscopic variceal ligation | 1.639 [1.203–2.234] | 0.002 |
| INR | 2.071 [1.641–2.615] | < 0.001 |
| Albumin | 0.486 [0.424–0.556] | < 0.001 |
| Platelet count | 1.001 [1.000–1.002] | 0.001 |
| Secondary malignant neoplasm (Respiratory and digestive organs) | 1.091 [0.853–1.396] | 0.489 |
| Secondary malignant neoplasm (other/unspecified) | 1.346 [1.051 to 1.725] | 0.019 |

AFP, alpha-1-fetoprotein; Atezo, atezolizumab; Bev, bevacizumab; CI, confidence interval; Dur, durvalumab; HR, hazard ratio; INR, international normalised ratio; Treme, tremelimumab

Atezo+Bev was used as the reference.
